# Effects of meaning and symmetry on judgments of size

**DOI:** 10.3389/fpsyg.2014.01270

**Published:** 2014-11-04

**Authors:** Rolf Reber, Bo T. Christensen, Beat Meier

**Affiliations:** ^1^Department of Psychology, University of OsloOslo, Norway; ^2^Department of Marketing, Copenhagen Business SchoolCopenhagen, Denmark; ^3^Department of Psychology, University of BernBern, Switzerland

**Keywords:** number processing, processing fluency, size perception, symmetry

## Abstract

Research has shown that people judge words as having bigger font size than non-words. This finding has been interpreted in terms of processing fluency, with higher fluency leading to judgments of bigger size. If so, symmetric numbers (e.g., 44) which can be processed more fluently are predicted to be judged as larger than asymmetric numbers (e.g., 43). However, recent research found that symmetric numbers were judged to be smaller than asymmetric numbers. This finding suggests that the mechanisms underlying size judgments may differ in meaningful and meaningless materials. Supporting this notion, we showed in Experiment 1 that meaning increased judged size, whereas symmetry decreased judged size. In the next two experiments, we excluded several alternative explanations for the differences in size judgments between meaningful and meaningless materials in earlier studies. This finding contradicts the notion that the mechanism underlying judgments of size is processing fluency.

## INTRODUCTION

How is physical size represented in the mind? The simplest answer would be that processing in the visual system results in a direct translation of physical size into mental size without any bias. Indeed, psychophysical experiments show that subjective length is a function of objective length (Stevens and Galanter, 1957). More recently, Casasanto and Boroditsky (2008) have observed an asymmetry between judgments of duration and judgments of size: Participants were unable to ignore irrelevant spatial information when making duration judgments. In their distance judgments, in contrast, participants were not biased by irrelevant duration information. This corresponds to the notion that people use spatial metaphors about time more often than temporal metaphors about space (e.g., Lakoff and Johnson, 1980). Taken together, these findings suggest that spatial judgments reflect reality quite well.

The positive correlation between physical size and mental size does not mean, however, that spatial judgments are always unbiased. For example, children from relatively poor economic backgrounds estimate coins as being larger than children from relatively rich economic backgrounds (Bruner and Goodman, 1947). The famous Ebbinghaus illusion shows that there are instances where size judgments are context-dependent (see Roberts et al., 2005). More recently, Reber et al. (2004b; Experiment 4; henceforth, the word study) have demonstrated a word-superiority effect for size judgments (see also New et al., in press). They briefly presented words and non-words that were composed of the same letters in four different font sizes, and participants had to indicate the font size on a rating scale. The main finding was that words were judged as being larger than non-words. Reber et al. (2004b) interpreted their finding as an effect of processing fluency – henceforth, fluency – which is the subjective ease with which information flows through the cognitive system. Fluency can be measured by measuring response times or by assessing subjective ratings of ease of identification (see Reber et al., 2004a; Wurtz et al., 2008). Reber et al. (2004b) argued that words are easier to process than non-words because words provide meaning and therefore facilitate encoding of the individual letters (see Reicher, 1969, for the word-superiority effect), and participants merely had to assess experienced processing fluency in order to make their size judgments. How would processing fluency translate into estimates of size? One possibility is that processing fluency is an indicator of how much information can be processed per time unit. As words, compared to non-words, activate a richer network of semantic nodes, more information per unit of time is processed. The ease of processing comes from top–down processing. A stimulus can be processed more easily if more information is available that allows the generation of concept-driven hypotheses about the stimulus elements, therefore processing more information totally and more information per unit of time.

By the same processing fluency logic, we would predict that symmetric forms would be judged to be larger than asymmetric forms. Symmetry provides information that allows the generation of hypotheses about the remaining elements of the stimulus. Through this predictability, a person can process more information per unit of time when a stimulus is symmetrical than when it is asymmetrical.

In a recent study, Reber et al. (2010; henceforth, the number study) examined comparative number magnitude judgments with symmetric and asymmetric numbers (such 66 versus 65). In their last experiment, they checked the effect of symmetry on font size judgments. A processing fluency account of size judgments (Reber et al., 2004b) would have predicted that participants judge symmetric numbers as having larger font size than asymmetric numbers because symmetric shapes can be more easily processed than asymmetric shapes (see Royer, 1981; Wurtz et al., 2008). Contrary to this prediction, however, asymmetric numbers were judged as having larger font size than symmetric numbers. As this last experiment was just a manipulation check, the authors did not discuss the effect further. Nevertheless, from the viewpoint of a fluency account, there exists a contradiction between the observation that words are judged as being larger than non-words, and the observation that symmetric numbers are judged as being smaller than asymmetric numbers.

There are at least two mechanisms that may underlie processing fluency: Symmetry, for example, may be processed more easily because it takes less time to process a stimulus when information is identical on both sides (see, Garner, 1974). This type of fluency is based on processing less information. If size judgments were based on amount of information instead of fluency, saving cognitive resources would translate into smaller stimulus size. Words, on the other hand, may be processed more fluently than non-words because they possess additional, semantic information that in turn would translate into larger stimulus size. Indeed, adding semantic information to non-words by attaching meaning to them in a reading training task led to reduced reading times (McKay et al., 2008). This suggests that integration of additional information facilitates word processing and thus increases fluency. Note that additional information needed to process asymmetric shapes does not have the same integrative function and therefore impairs fluency, compared to symmetric shapes that do not convey meaning. Thus, and in contrast to the claim by Reber et al. (2004b), some other variable than processing fluency may account for size judgments.

However, the two studies employed different materials and slightly different methods and can therefore not be taken as evidence in favor of an amount of information account. We addressed four such differences in the current experiments. First, there were as many words as non-words in the word study, but there were five symmetric numbers and 26 asymmetric numbers in the number study. In order to exclude effects of proportion of stimuli, we manipulated this variable in Experiment 2: In the first condition, asymmetric numbers were shown more often than symmetric numbers, as in the number study. In the second condition, the two types of numbers were shown equally often, as words and non-words in the word study. In the third condition, symmetric numbers were shown more often than asymmetric numbers. Although using more asymmetric numbers than symmetric numbers was appropriate for the manipulation check in the number study, it may have biased font size judgments; a problem we circumvented in the current study.

Second, a backward pattern mask was shown in the word study, but not in the number study. We therefore presented in Experiment 2 numbers followed by a mask in order to assert that font size judgments in numbers can be observed if a mask is shown, as in the word study.

Third, words in the word study (Reber et al., 2004b) had five letters, whereas numbers in the number study (Reber et al., 2010) had two digits. We explored this issue in Experiment 3 where we examined symmetry effects on font size judgments for numbers with five digits (which is as many digits as the number of letters in the word study).

**FIGURE 1:**
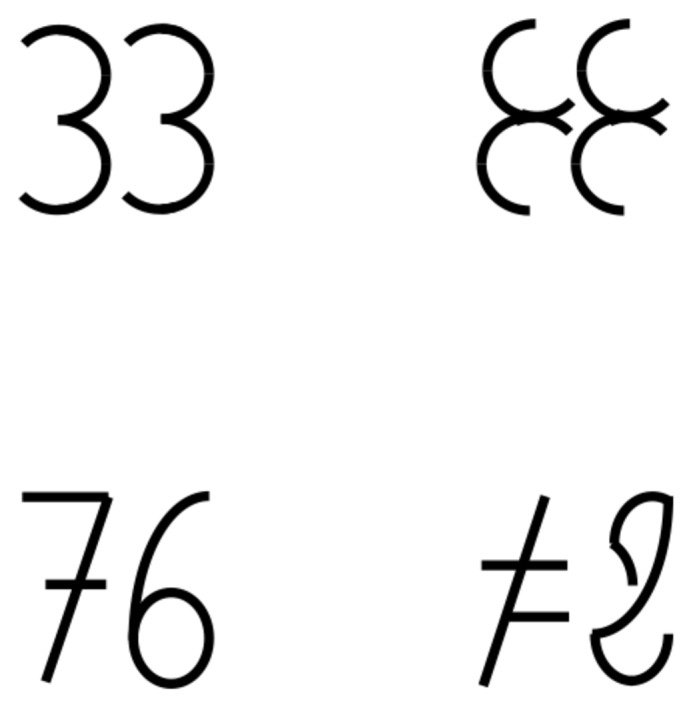
**Materials used in Experiment 1.** On the left panel are numbers, on the right panels the corresponding meaningless characters. The digit 7 is written in the notation used for handwriting in many European countries (with a dash in the center).

Before we examine these methodological issues, we address the difference between meaningful and meaningless stimuli first. In Experiment 1, we examined the amount of processed information account more directly by manipulating meaningfulness and symmetry within the same experiment. To this purpose, we constructed meaningless characters that were composed of elements of numbers (see **Figure 1**). This resembled the method used in the word study where each non-word consisted of the same letters as a corresponding word and served the purpose to display the same visual elements in the two meaningfulness conditions. We predicted that asymmetric characters – regardless of meaningfulness – were judged as having larger font size than symmetric characters, as in the number study, and that numbers are judged as having larger font size than meaningless characters, analogous to the word study.

## EXPERIMENT 1

We wanted to test in a single experiment whether the two factors isolated in the former studies and experiments indeed affect judged size: symmetry and meaningfulness. Symmetry was manipulated by translational symmetry of numbers whereas meaning was manipulated by presenting numbers or meaningless characters. In this experiment, we examine whether the effects of symmetry found in the different number experiments can also be observed with meaningless materials.

### METHOD

#### Participants

Fifty-five undergraduate students from the University of Bergen (33 female, 22 male) participated in the experiment for payment. Mean age was *M* = 22.42 (SD = 2.12) years.

#### Materials

We used four symmetric and four asymmetric two-digit numbers. The numbers used in the experimental trials were: 33, 44, 66, 77, 34, 43, 67, and 76. In addition to the numerical stimuli, we constructed meaningless characters that were assembled from parts of the digits of the numerical stimuli (see **Figure 1**). Like in Reber et al. (2010), all the symmetrical items possessed translatory symmetry, which is the repetition of an element without changing its vertical position, AB ↔ AB, but not vertical symmetry, which is the mirroring of element along a vertical axis, AB ↔ BA (see Weyl, 1952). Each number and each character pair was drawn in four different sizes, with stimulus width and stimulus height being identical, 34, 36, 38, or 40 mm (1.9, 2.1, 2.2, or 2.3°). This yielded 4 (stimulus) × 2 (meaning) × 2 (symmetry) × 4 (size) = 64 experimental trials.

In order to render participants familiar with the size of the stimuli, we presented 32 practice trials before the experimental trials started. Numbers used in the practice trials were, 88, 22, 28, and 82, respectively, or the corresponding characters.

#### Procedure

The participants were seated in front of a computer monitor and given information about the purpose of the experiment. They were told that we are studying visual judgment and asked to give their consent to participate in the experiment by pressing a key on the keyboard. The participants then were instructed to rate the size of the numbers on a scale from 1 (small) to 9 (big), using the number keys on the keyboard (as in Reber et al., 2004b).

Participants were first presented with a black fixation cross on a white background for 500 msec in order to focus their attention on the center of the screen. Then a stimulus, symmetric or asymmetric, and consisting of digits or characters, was presented for 200 msec in the center of the screen. The participants had to judge how big the target item was on the 9-point scale. The order of the numbers was random.

### RESULTS AND DISCUSSION

#### Size judgments

For each participant, we computed the mean rating for each of the meaningfulness × symmetry × font size conditions. Means and standard deviations for ratings are listed in **Table 1**. We performed a 2 × 2 × 4-factorial ANOVA, with all variables, meaningfulness (number; meaningless characters), symmetry (symmetric versus asymmetric), and font size (34, 36, 38, 40 mm) manipulated within subjects. Not surprisingly, participants were able to distinguish between the different font sizes, *F*(3,162) = 120.66, *p* < 0.001. We also replicated the effect of symmetry on perceived font size observed in the number study; participants perceived symmetric numbers as being smaller than asymmetric numbers, *F* (1,54) = 63.73, *p* < 0.001, *r_e_* = 0.74. The main effect of meaning was also significant, *F*(1,54) = 40.60, *p* < 0.001, *r_e_* = 0.66, indicating that the font size of number was judged as larger than the font size of meaningless characters. Two interactions were significant, the two-way interaction between meaning and size, *F*(3,162) = 8.01, *p* < 0.001 and the three-way interaction between meaning, symmetry, and size, *F*(3,162) = 12.32, *p* < 0.001. The other effects were not significant, *Fs* < 2.05, *p*s > 0.15.

**Table 1:** Means and standard deviations (in parentheses) for font size ratings for symmetric and asymmetric numbers, and numbers versus character pairs (Experiment 1).

Proportion	Size	Symmetric	Asymmetric
**Numbers**
	34 mm	5.58 (1.21)	6.21 (1.19)
	36 mm	6.17 (1.17)	6.48 (1.32)
	38 mm	6.93 (1.17)	7.17 (1.28)
	40 mm	7.22 (1.23)	7.54 (1.24)
**Character pairs**
	34 mm	5.25 (1.18)	5.27 (1.20)
	36 mm	5.63 (1.22)	6.02 (1.17)
	38 mm	6.44 (1.15)	6.73 (1.13)
	40 mm	6.89 (1.15)	7.29 (1.13)

We further analyzed the two-way interaction by calculating *t*-tests for the difference between numbers and characters for each size. All differences were significant, *t*s (54) between 3.57 and 6.76, but the effect size r for the largest font size (*r_e_* = 0.45) was smaller than for the smaller font sizes (*r_e_*s between 0.59 and 0.68), indicating that the interaction is due to the smaller difference between numbers and meaningless characters for the largest font size than for the smaller font sizes.

We also calculated eight *t*-tests between asymmetric and symmetric stimuli in order to examine the three-way interaction. All of these *t*-tests were significant at the Bonferroni-corrected 0.00625 level [*t*s (54) between 3.02 and 6.98, *r_e_*s between 0.38 and 0.69], with the exception of the difference between symmetric and asymmetric character pairs in the smallest font condition, *t*(54) = 0.212. This pattern has been observed in the word study where there was no difference between words and non-words for the smallest font size. They explained this effect with the fact that there are two mappings of judged font size as a function of real font size, one for words and one for non-words. If the linear function for font size of words increases by a greater slope than the linear function for font size of non-words, and both functions pass the same origin (assuming that 0 size being judged as 0), it is obvious that the smallest font size shows the smallest difference in judged font size. In this experiment, however, we observed this effect only for meaningless characters, not for numbers. If this finding is stable, it may mean that different mechanisms underlie the processing of meaningless characters and numbers. As this issue was outside the focus of this paper, we did not follow up this result.

We found an effect of both symmetry and of meaningfulness in the direction observed before in the word study and in the number study. Asymmetric characters were judged as having larger size than symmetric characters, and meaningful numbers were judged as having larger size than meaningless characters derived from the number stimuli. In sum, this finding suggests that amount of processed information, not fluency, influences font size judgments.

#### Correlations between judged font size and number magnitude

In line with earlier research on size congruence effects between number magnitude and font size in comparative number judgments (Besner and Coltheart, 1979; Henik and Tzelgov, 1982), we expected that greater numbers were seen as bigger in physical size. We did not expect such an effect for meaningless characters. We computed the correlations between numerical magnitude and physical size across the eight stimuli, separately for numbers and for meaningless characters. Our predictions were confirmed. For numbers, the correlation was positive and significant, *r* = 0.73, *p* = 0.040; for meaningless characters, the correlation was close to 0, *r* = 0.02. This finding suggests that participants processed the meaning of the numbers, but magnitude of the numbers from which the characters were made was unrelated to judged font size. The finding also serves as a manipulation check that the meaningless characters did not bear any resemblance to the number they were constructed of.

After we have shown that processing fluency cannot account for judgments of size, we address in the next two experiments methodological differences between the word study and the number study in order to exclude other potential explanations of the different findings.

## EXPERIMENT 2

We addressed two possible differences in the earlier studies, the proportion of symmetric and asymmetric numbers and backward masking. We manipulated the proportion of symmetric and asymmetric numbers systematically and introduced a mask, thus creating the same conditions as in the word study, where there were as many words as non-words, and where a pattern mask was presented after the stimuli.

### METHOD

#### Participants

Fifty-seven undergraduate students from the University of Bergen (42 female, 15 male) participated in the experiment for payment. Mean age was *M* = 21.02 (SD = 2.01) years. There were three experimental conditions with 19, 20, and 18 participants, respectively: (1) more symmetric than asymmetric stimuli; (2) as many symmetric as asymmetric stimuli; (3) fewer symmetric than asymmetric stimuli.

#### Materials

We used the same numbers as in Experiment 1: 33, 44, 66, 77, 34, 43, 67, and 76. In contrast to Experiment 1, we did not present meaningless stimuli. The numbers were shown, one by one, in the center of the screen in four different sizes: 15, 16, 17, or 18 points. Numbers were about 7, 7, 8, and 8 mm high (visual angle 0.4 and 0.5°), and about 11, 12, 12, and 13 mm wide (visual angle 0.6, 0.7, 0.7, and 0.7°). Each number was immediately followed by a gray mask presented for 200 ms in order to prevent an afterimage. The gray mask was 37 mm wide (visual angle 2.1°) and 17 mm high (visual angle 1.0°). Depending on the condition a participant was assigned to, they were exposed to a different ratio of symmetric and asymmetric numbers. There were 128 trials in each condition. In the condition with more symmetric than asymmetric numbers, each symmetric number was presented six times and each asymmetric number was presented twice in each of the four font sizes. This resulted in 96 (4 numbers × 6 presentations × 4 sizes) trials with symmetric numbers and 32 (4 × 2 × 4) trials with asymmetric numbers. In the condition with less symmetric than asymmetric numbers, each symmetric number was presented twice and each asymmetric number was presented six times in the same size. This resulted in 32 trials with symmetric numbers and 96 trials with asymmetric numbers. In the condition where symmetric and asymmetric numbers were presented equally often, each number was shown four times in the same size. This resulted in 64 (4 numbers × 4 presentations × 4 sizes) trials with symmetric numbers and as many trials with asymmetric numbers. Again, participants were given practice trials in order to give them an idea of how large the stimuli were. We presented 32 practice trials before the experimental trials started. Numbers used in the practice trials were: 88, 22, 28, 82, and each number was shown twice in the same size. Participants were randomly assigned to the three different conditions.

#### Procedure

The procedure was the same as in Experiment 1.

### RESULTS AND DISCUSSION

#### Size judgments

The means and standard deviations for the different conditions can be seen in **Table 2**. The experimental setup resulted in a 3 × 2 × 4-factorial design. The first factor represented the three different conditions for the proportion of symmetric and asymmetric numbers, manipulated between-subjects. The other two factors were symmetry versus asymmetry and font size, manipulated within-subjects.

**Table 2:** Means and standard deviations (in parentheses) for font size ratings for symmetric and asymmetric numbers, depending on proportions of symmetric and asymmetric stimuli, with mask (Experiment 2).

Proportion	Size	Symmetric	Asymmetric
**Sym < Asym (*n* = 19)**
	15 pt	4.73 (1.11)	4.76 (1.08)
	16 pt	4.74 (1.15)	5.09 (0.98)
	17 pt	5.78 (0.80)	5.93 (0.87)
	18 pt	6.83 (0.98)	7.11 (1.12)
**Sym = Asym (*n* = 20)**
	15 pt	4.88 (1.45)	5.07 (1.41)
	16 pt	5.14 (1.48)	5.19 (1.41)
	17 pt	5.95 (1.38)	6.43 (1.44)
	18 pt	7.04 (1.56)	7.18 (1.69)
**Sym > Asym (*n* = 18)**
	15 pt	4.79 (1.15)	5.00 (1.28)
	16 pt	5.08 (1.09)	5.20 (1.00)
	17 pt	6.05 (1.15)	6.32 (1.10)
	18 pt	7.07 (1.11)	7.29 (1.39)

Participants were able to distinguish between the different font sizes, *F*(3,162) = 118.17, *p* < 0.001. More importantly, participants perceived asymmetric numbers as having larger font size than symmetric numbers, *F*(1,54) = 58.63, *p* < 0.001, *r_e_* = 0.76. The other effects were not significant, *Fs* < 1.98, *ps* > 0.07. The symmetry effect appears regardless of the proportion of symmetric and asymmetric stimuli, and regardless of whether the numbers were presented without mask, as in the number study (Reber et al., 2010) and in Experiment 1, or with mask, as in this experiment. The latter observation means that the effects in the word study by Reber et al. (2004b) did not depend on presenting a mask.

#### Correlations between judged font size and number magnitude

As in Experiment 1, we computed correlations between the judged font size and numerical magnitude across the eight stimuli, averaged across the four font sizes. Indeed, this correlation was positive and significant, *r* = 0.91, *p* = 0.002). Again, participants processed the meaning of the numbers, and number magnitude influenced font size judgments.

## EXPERIMENT 3

Whereas the words in the word study consisted of five letters, the numbers used in the number study had two digits. In order to examine the possibility that effects of symmetry were moderated by the length of a string, we conducted Experiment 3 where we presented five-digit strings instead of two-digit strings like in the earlier number study.

### METHOD

#### Participants

Seventeen undergraduate students from the University of Bergen (15 female, 2 male) participated in the experiment for payment. Mean age was *M* = 20.12 (SD = 1.60) years.

#### Materials

Stimuli used in the experiment were 31 symmetric and asymmetric numbers that were five digits long. We used numbers between 30000 and 79999. Whereas the 26 asymmetric numbers were composed of five different digits, the five symmetric numbers were 33333, 44444, 55555, 66666, and 77777. We presented 20 (4 times 5) symmetric numbers and 104 (4 times 26) asymmetric numbers, or 124 trials totally. Beyond symmetry, font size was an independent variable. The five-digit numbers were about 30, 31, 32, and 34 mm wide (visual angle 1.7, 1.8, 1.8, and 1.9°) and about 7, 7, 8, and 8 mm high (visual angle 0.4, 0.4, 0.5, and 0.5°). Before the experimental trials began, participants were given 12 practice trials (not included in the analysis) with five digit numbers ranging either from 10000 to 29999 or from 80000 to 99999.

#### Procedure

The procedures were the same as in Experiment 1.

### RESULTS AND DISCUSSION

#### Size judgments

For each participant, we computed the mean rating for each of the symmetry × font size conditions. Means for ratings are shown in **Table 3**. The experimental setup resulted in a 2 × 4-factorial ANOVA design. The first factor represented symmetric versus asymmetric numbers, and the second factor represented the four presented font sizes (15, 16, 17, 18 points); both factors were manipulated within-subjects. Not surprisingly, participants were able to distinguish between the different font sizes, *F*(3,48) = 57.31, *p* < 0.001. More importantly, participants perceived asymmetric numbers as having larger font size than symmetric numbers, *F*(1,16) = 5.57, *p* = 0.031, effect size *r_e_* = 0.51. The symmetry × font size interaction was not significant, *F*(3,16) = 0.02, *p* = 0.99. Like the two-digit numbers, five-digit numbers showed a symmetry effect, suggesting that the symmetry effect on font size judgments does not depend on string length.

**Table 3:** Means and standard deviations (in parentheses) for font size ratings for symmetric and asymmetric numbers, displayed for 200 ms, five digit numbers (Experiment 3).

Size	Symmetric	Asymmetric
15 pt	4.33 (1.14)	4.50 (0.96)
16 pt	4.49 (1.09)	4.69 (0.98)
17 pt	5.60 (1.25)	5.75 (1.09)
18 pt	6.56 (1.50)	6.75 (1.20)

*n = 17.*

#### Correlations between judged font size and number magnitude

We again computed correlations between the judged font size and numerical magnitude across the 31 stimuli, averaged across the four font sizes. Indeed, this correlation was positive and significant, *r* = 0.78, *p* < 0.001. Participants indeed processed the meaning of the numbers, and number magnitude influenced font size judgments.

## GENERAL DISCUSSION

Despite the fact that judged size corresponds quite well with real size, context manipulations bias size judgments, as shown by the Bruner and Goodman (1947) study and by the Ebbinghaus illusion. The starting point of the present study was the finding in the number study by Reber et al. (2010) that asymmetric numbers were judged as having larger font size than symmetric numbers. This observation contradicted the fluency interpretation provided by Reber et al. (2004b) to account for their observation that words were judged as having larger font size than non-words. By manipulating symmetry and meaning orthogonally within the same experiment (Experiment 1), we could show that it is not processing fluency that drives the effect. However, before rejecting a fluency account, we had to examine whether methodological differences between the two studies – the word study and the number study – could account for the findings that were contradictory from the viewpoint of a fluency account. We showed that this was not the case (Experiments 2 and 3).

The correlations between number magnitude and judged font size were always positive and significant when numbers were presented, but not for meaningless characters made from numbers. This shows that our manipulation of meaning was effective in that the participants processed the meanings of the number, and that our characters made up from numbers did not bear numerical meaning.

Despite the clear-cut results, one limitation has to be acknowledged. The amount of processed information has neither been defined properly nor measured directly but inferred from characteristics of the materials. Although it is plausible from both semantic network accounts of the processing of meaning (e.g., the spreading activation account by Collins and Loftus, 1975) and from informational accounts of form perception (Garner, 1974), future research may directly assess the amount of information that has to processed for the different classes of stimuli. Only then can we begin to explore alternative explanations after the fluency account has been falsified.

In sum, we have shown that the mechanism underlying size judgments is not processing fluency. Further research has to properly operationalize and measure amount of information in order to test the plausible assumption that judgments of size depend on how much information has to be processed and not on ease of processing. This finding opens up new avenues for research on judgments of size, of duration, and for the relationship between amount of information and metacognitive experiences, such as processing fluency.

## Conflict of Interest Statement

The authors declare that the research was conducted in the absence of any commercial or financial relationships that could be construed as a potential conflict of interest.
